# Symptoms, coronary artery disease and percutaneous coronary intervention: connecting the dots

**DOI:** 10.1097/HCO.0000000000001246

**Published:** 2025-09-10

**Authors:** Shayna Chotai, Kayla Chiew, Rasha Al-Lamee

**Affiliations:** a National Heart and Lung Institute, Imperial College London; b Imperial College Healthcare NHS Trust, London, UK

**Keywords:** angina, coronary artery disease, percutaneous coronary intervention, symptoms

## Abstract

**Purpose of review:**

Symptom relief is now recognized as the primary remit of percutaneous coronary intervention (PCI) in patients with stable coronary artery disease. The relationship between the nature of angina symptoms and the likelihood of successful symptom relief from PCI had not been systematically studied until recently.

**Recent findings:**

The ORBITA-2 symptom-stratified analysis found that while the severity and nature of symptoms were poorly associated with the severity of coronary disease, the nature of the symptoms powerfully predicted the efficacy of PCI in relieving angina. Specifically, patients with typical or “Rose angina” were most likely to benefit from PCI, while those with atypical symptoms were less likely to see significant improvement beyond placebo. Furthermore, the ORBITA-STAR study demonstrated that patients whose angina symptoms closely matched those induced by balloon occlusion at the site of a coronary stenosis were significantly more likely to experience symptom relief from PCI.

**Summary:**

Symptom analysis offers a powerful tool for predicting the efficacy of PCI. Misattributing noncardiac symptoms as angina often results in ineffective intervention, highlighting the critical importance of accurate and thoughtful symptom assessment, particularly in identifying typical angina. The persistent challenge of residual angina despite technically successful PCI reflects not a failure of the intervention itself, but a shortcoming in diagnostic precision to identify those who will benefit. Future research should focus on refining clinical predictors to better guide the selection of patients most likely to benefit from revascularization.

## INTRODUCTION: ANGINA TO INTERVENTION

In the 1970s, Andreas Grüntzig pioneered the concept and development of coronary angioplasty with one clear and straightforward goal: to alleviate the symptoms of angina that limited the lives of many patients [1]. Decades later patients still present with angina that restricts daily activities, diminishes quality of life, and drives individuals to seek medical care. Since Grüntzig's time, the interventional cardiology community has hoped that coronary interventions could do more than simply relieve symptoms. Driven by the belief that these procedures could contribute further, clinical trials were designed to evaluate so-called “hard” endpoints – myocardial infarction and death – on the premise that meaningful interventions must save lives. Yet, after several landmark trials [2,3], we learned that percutaneous coronary intervention (PCI) in patients with stable angina does not reduce cardiovascular event rates or improve survival. Far from undermining the value of PCI, these findings invite us to reconsider an outdated hierarchy of outcomes that treats symptom relief as secondary or “soft.” Symptoms matter deeply to patients. Our colleagues in orthopaedics perform joint replacements not to extend life expectancy, but to restore quality of life; a goal that is both meaningful and measurable. Similarly, we should not underestimate the impact of relieving angina. Perhaps it is time to re-centre our focus on what patients truly value: the ability to live with less pain and improved daily function. Symptom relief is not a consolation prize, it is the primary goal of PCI in stable coronary disease.

However, the assessment of symptoms comes with its own complexity. Angina was first described in the 18th Century [4]. Doctors are taught early in medical school that history taking is the bedrock of the diagnosis. We understand that pain of a cardiac nature usually presents as a squeezing or tight sensation in the chest and can be associated with radiation to the jaw and/or left shoulder. In cases of stable angina, this sensation is brought on by exertion and relieved by rest. Despite this teaching, somewhere along the line, we seem to lose this attention to symptomatology. Unfortunately, clinical presentations do not always conform to textbook definitions. For patients with atypical symptoms, we are called upon to make a diagnosis and are under pressure to treat the symptoms. We may accept atypical symptoms as justification to order a cardiac investigation, and the subsequent results become our primary focus. When significant coronary artery disease (CAD) is found, we rarely look back at the presenting symptoms to decide on treatment. In 2025, the primary tool used in clinic to diagnose “angina” is no longer the history, but the myriad of anatomical and functional investigations for CAD now available to us. Has this shift away from prioritization of symptoms had an impact on the success of our treatments? What is the relationship between symptoms, and the severity of disease measured by these tests? And does this matter? 

**Box 1:**
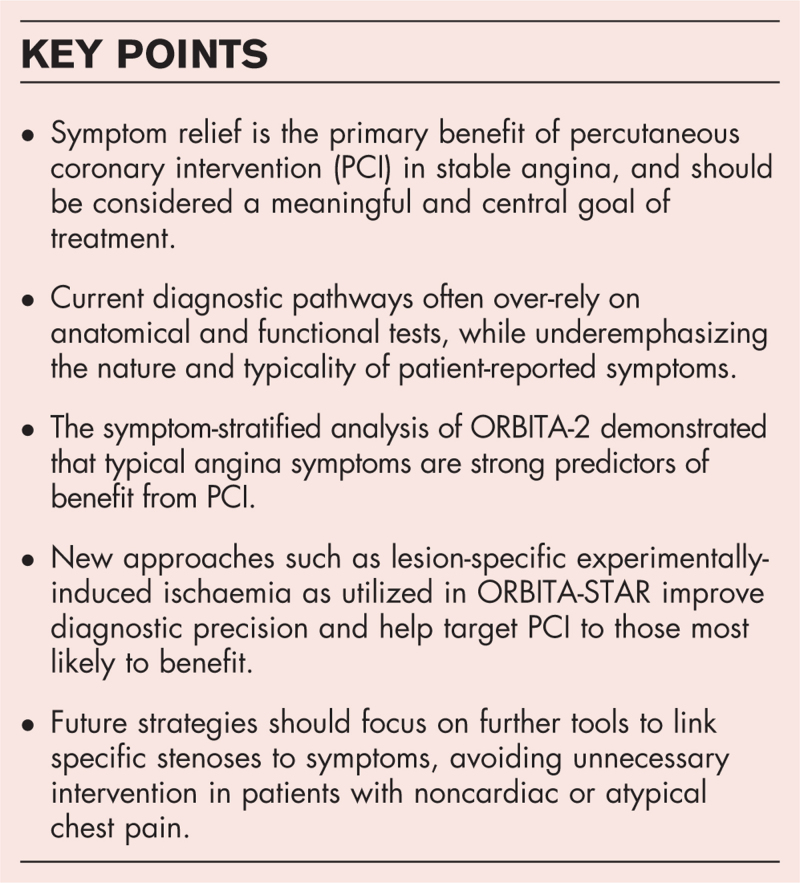
no caption available

## SYMPTOM RELIEF WITH PERCUTANEOUS CORONARY INTERVENTION

Historical trials of PCI in stable CAD have shown large symptom relief benefits with PCI, albeit with the lack of placebo-control and blinding [5–7]. We know that the evaluation of symptoms, a subjective endpoint, without blinding, is not without significant limitation, especially in the context of a procedure or intervention, where the placebo effect has been shown to be greatly pronounced [8]. ORBITA [9] was the first placebo-controlled trial of PCI and showed that PCI improved angina much less than expected when compared to placebo. Importantly, it demonstrated the feasibility and necessity of judging symptoms after PCI with blinded placebo-control.

However, residual angina is common after PCI, and continues to be a clinical challenge. It is reported to be as high as 60% despite technically successful PCI in blinded studies [9,10^▪^] and up to 40% in unblinded studies [11]. Perhaps the underlying issue stems from our selection of patients. In recent years, the definition of angina has broadened to acknowledge the subjective nature of symptom reporting and the influence of sex, age, ethnicity, and certain comorbidities such as diabetes. The recent evolution of this definition to encompass atypical symptoms or angina-equivalents has resulted in a more heterogeneous cohort in the catheterization laboratory, many of whom may not have angina and therefore have little to gain from PCI. Indeed an analysis of SCOTHEART, a large United Kingdom cohort with suspected angina, found that females were more likely to have atypical symptoms and normal or unobstructed coronary arteries on computed tomography coronary angiography (CTCA) compared to males, resulting in higher rates of reclassification of symptoms as noncardiac. [12] Clearly, improving procedural success from PCI will likely depend on increasing diagnostic accuracy of angina and the careful selection of patients to whom we offer revascularization.

## TYPICALITY OF ANGINA MATTERS

The powerful ability of symptoms to predict response to PCI should not be underestimated. Unblinded trials suggested that those with the highest burden of angina benefited the most from PCI [5,7]. Whilst they evaluated symptom frequency, burden, and the impact on quality of life, the *nature* of symptoms, in particular *typicality,* has not routinely been assessed. This was a key feature of the ORBITA-2 trial [10^▪^], in which Rose angina [13] and guideline-described typicality were incorporated within symptom assessment. The Rose Angina Questionnaire categorizes symptoms as Rose angina, similar to conventionally termed ‘typical’ angina, or Rose nonangina.

In ORBITA and ORBITA-2, patients with stable angina and coronary artery disease were randomized to undergo either PCI or a placebo procedure. To maintain blinding, patients in the catheterization lab wore over-the-ear headphones with music for auditory isolation and received benzodiazepines and opioids to achieve deep conscious sedation. Blinding was preserved throughout the follow-up period. In ORBITA, all patients received optimized antianginal therapy, allowing the trial to assess the added benefit of PCI. In contrast, ORBITA-2 evaluated the effect of PCI without background antianginal medications, eliminating potential attenuation from pharmacologic therapy. Additionally, ORBITA-2 differed by placing greater emphasis on symptom assessment, using a patient-centred and sensitive primary endpoint – a daily symptom score – rather than exercise treadmill time.

The symptom-stratified analysis of ORBITA-2 demonstrated the importance of identifying ‘typical’ angina and its ability to differentiate patients who are most likely to achieve symptom relief from PCI [14^▪▪^]. Firstly, it found that the nature of symptoms was a strong predictor of the placebo-controlled efficacy of PCI. Patients with guideline-based ‘typical’ or ‘Rose’ angina had almost twice the odds of being symptom-free after PCI compared to those with atypical symptoms. Interestingly, disease severity as assessed by quantitative coronary angiography or intracoronary physiology, was poorly associated with symptom severity. Furthermore, there was little relationship between sex or diabetes, and Rose angina; two key demographic features that are widely believed to influence the presentation of angina. As we continue to develop our understanding of the complex relationship between stenoses, symptoms and the role of revascularization, we are reminded that it is patients with typical angina that stand to gain the most from PCI.

## POTENTIAL MODIFIERS OF TREATMENT EFFECT

Further data emerged from subsequent secondary analyses that provided insight into the clinical investigations that may augment treatment benefit of PCI. In the physiology-stratified analysis of ORBITA-2, stenosis severity, as assessed by fractional flow reserve (FFR) and instantaneous wave-free ratio (iFR), were found to be good predictors of symptom relief with PCI [15^▪^] in patients not taking antianginal medications. Patients with the lowest quartile of FFR and iFR had the greatest placebo-controlled improvement in angina symptom score. This interaction was modified by symptom characteristics. In patients with Rose nonangina, much lower physiological values were needed to confer symptom benefit with PCI, lending support to the value of assessing typicality of symptoms. Notably, the relationship between physiological severity and treatment effect was not so clear in ORBITA. Here, lower FFR and iFR values predicted stress echocardiography ischaemia improvement with PCI, but there was no association between physiology and symptom relief. This may have been due to the smaller sample size and the attenuating impact of intense antianginal medication which may have dissociated the link between symptoms and physiology [16].

In both ORBITA studies, stress echocardiography ischaemia predicted symptom relief from PCI. The greater the degree of ischaemia quantified by DSE score, the larger the improvement in angina symptom score from PCI. [17,18^▪^] This effect was less pronounced in ORBITA compared to ORBITA-2, with a relationship seen in only one symptom endpoint, patient-reported angina frequency.

Finally, patterns of coronary artery disease, namely focal or diffuse, may be an important consideration when planning a treatment strategy for patients with angina. Unblinded studies of PCI have shown superior procedural outcomes in focal compared to diffuse disease, achieving greater minimum luminal areas and higher post-PCI physiological indices [19,20]. The impact of disease pattern may also influence symptom endpoints, with lower rates of residual angina and greater improvement in quality of life in focal compared to diffuse disease [11,21]. The placebo-controlled, blinded impact of disease pattern on symptom relief from PCI however is less clear. In a disease pattern-stratified analysis of ORBITA, whilst PCI in focal disease resulted in a greater reduction in stress echocardiography ischaemia, there was no differential impact on symptoms [22]. More data are awaited from ORBITA-2 that will further advance our understanding of the relationship between pattern of disease and symptom relief with PCI.

## VERIFICATION OF ANGINA IN THE CATHETERISATION LABORATORY

Prediction of symptom relief with PCI is an aspirational goal. Perhaps we can go one step further in this era of precision medicine and determine more directly, which patients truly have symptoms derived from their coronary stenosis to target PCI to those who are most likely to derive benefit. Many asymptomatic patients have visually and functionally severe stenoses picked up incidentally. On the other hand, some patients with a far lower burden of disease are highly symptomatic. We are starting to understand that the relationship between symptoms, anatomy and ischaemia is highly individual, and may not be as linear as we believe.

ORBITA-STAR, an *n*-of-1 experimental placebo-controlled study aimed to map individual symptoms directly to a stenosis using an ischaemic stimulus in the catheterization laboratory [23^▪▪^]. Participants with stable angina undergoing single-vessel PCI underwent episodes of placebo-controlled low-pressure balloon occlusion across their “culprit” stenosis. Patients were asked to report the similarity of their experimentally-induced symptoms to their usual angina. The similarity score ranged from −10 (placebo replicated the symptom more than balloon occlusion) to +10 (balloon occlusion exactly replicated the symptom). The primary endpoint was the ability of the similarity score to predict symptom relief with PCI.

**FIGURE 1:**
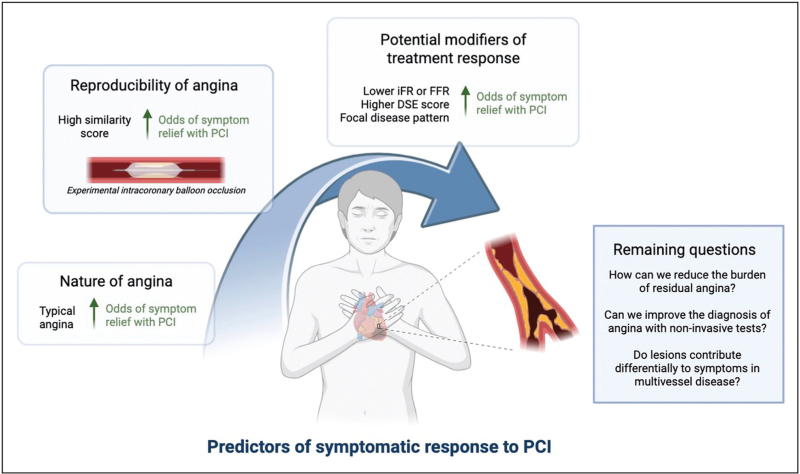
Current evidence for predictors of symptomatic response to percutaneous coronary intervention: findings from ORBITA-2 and ORBITA-STAR. DSE, dobutamine stress echo; FFR, fractional flow reserve; iFR, instantaneous wave-free ratio; PCI, percutaneous coronary intervention.

This placebo-controlled similarity score was shown to be a strong predictor of symptom improvement with PCI. Those whose presenting symptoms were replicated by experimentally-induced ischaemia at the location of the stenosis, were more likely to report symptom relief with PCI. In patients with high similarity scores, there was almost complete eradication of symptoms with PCI. These intuitive results demonstrated for the first time that, even in patients with anatomically and physiologically severe disease, symptom relief with PCI was not universal. It is clear that in some patients, epicardial coronary disease does not necessarily result in angina, and their presenting symptoms may not be related to their stenosis. Despite the availability of increasingly sensitive tests for coronary artery disease and ischaemia, it appears that thorough symptom analysis remains more pertinent than ever (Fig. 1).

## REPORTING AND RECORDING ANGINA

Perhaps it is not only important to ask the right questions, but also to consider the *way* in which we ask them, to improve the diagnostic accuracy of angina assessment in clinical practice. This will be particularly important if we elevate angina to a primary endpoint in clinical trials. To standardize the reporting of angina and enable comparability between trials, a number of validated questionnaires such as the Seattle Angina Questionnaire (SAQ) [24] are commonly used. However, these single-point-in-time assessments lack sensitivity to capture the variability of day-to-day symptoms, and importantly do not acknowledge adaptations to activity and behavioural change that patients may make in response to symptoms. For example, if a patient experiences symptoms each time they play tennis, they may avoid the activity and report no angina, leading to an underestimation of symptom burden by the questionnaire. Furthermore, whether patient-reported, such as the SAQ, or physician-assessed, such as the Canadian Cardiovascular Society (CCS) class [25], all approaches are liable to significant observer and recall bias [26].

The gold standard assessment of angina is to directly ask patients to report their symptoms everyday. In the past, this was performed using a paper angina diary. However, this also is limited by the need to remember to contemporaneously fill in the diary every day. The ORBITA-app was developed to overcome some of these barriers [27]. As a smartphone-based application, this method of daily symptom assessment enables the capture of temporal data with high sensitivity. Notably, the app was developed in consultation with patients living with angina, and the patient-centred questions and user interface reflect this. At the point of onboarding, patients are asked to describe their usual angina and the types of activities that usually trigger it. They are then asked to report the presence or absence of symptoms everyday. Once a week, they are also asked if they had symptoms when performing the preselected individualized precipitating activities. An advance on standard paper-based daily angina diaries, the app enables longer periods of symptom assessment above the typical 7-day period, and facilitates reminder prompts to maximize completeness of data collection. The ORBITA-app has successfully been used in a number of clinical trials with high compliance rates above 99% for up to 6 months of follow-up [28].

## CONCLUSION: USING SYMPTOMS AS A DIAGNOSTIC TOOL

We need to reflect on how often, we as clinicians, recommend coronary intervention primarily based on patient symptoms. In today's practice, we lean heavily on anatomical imaging and ischaemia testing to diagnose angina and identify candidates for PCI. Yet, growing evidence suggests these investigations are poor surrogates for what we ultimately aim to treat: symptoms. Once significant anatomy or ischaemia is detected, the clinical focus tends to shift almost entirely to treating the stenosis – often regardless of whether the symptoms were actually typical of angina. This creates further problems. When noncardiac chest pain is mislabelled as angina, we set PCI up to fail. When our PCI disappointingly does not relieve symptoms, we embark on a fishing expedition to search for an alternative cardiac cause. In some patients, this may be concomitant microvascular disease, however in many, particularly those with atypical symptoms, it may be that there was never a cardiac cause.

**FIGURE 2:**
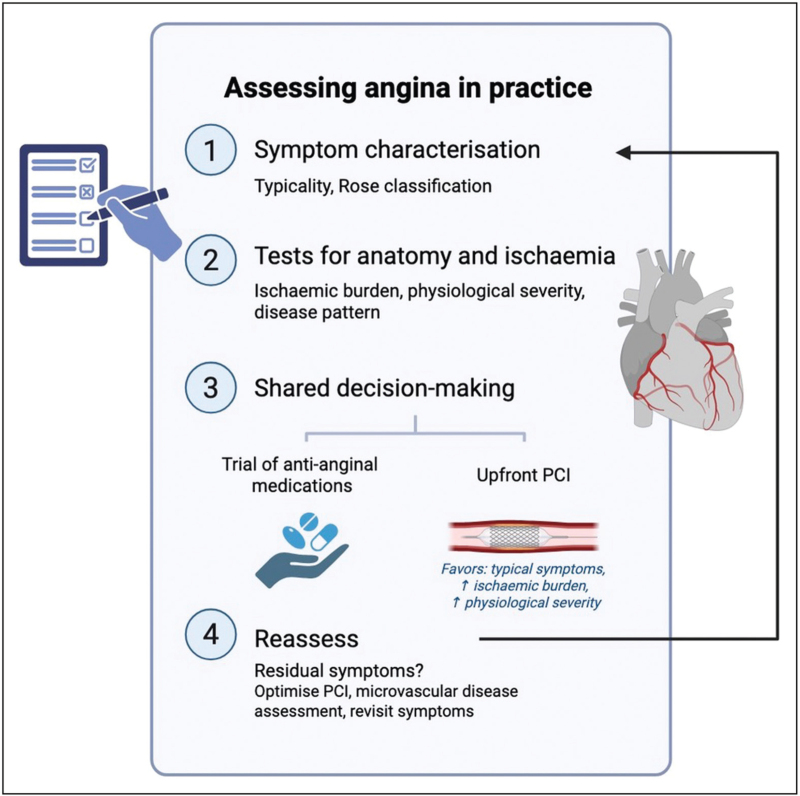
Suggested algorithm for the assessment of patients referred with angina. PCI, percutaneous coronary intervention.

Placebo-controlled trials have made a compelling case for returning to fundamentals. Systematic and thoughtful symptom evaluation should be used as a diagnostic tool to guide our decision-making. The first aim in clinic should be to evaluate whether the character of the symptom is typical of angina or not. We must remember that it is not the frequency or severity of symptoms that matter, but the nature of symptoms that predicts the efficacy of our ischaemia-relieving treatment. If a patient does not exhibit typical angina, they are unlikely to benefit from PCI – even in the presence of significant coronary artery disease. Of course these individuals require guideline-directed medical therapy for risk reduction, but we should recognize that intervention will not offer symptom relief. Conversely, if a patient has typical symptoms, on top of risk reduction medications, PCI can offer symptom relief. Additional testing for ischaemia can also help to stratify the likely response to PCI, particularly in those with atypical symptoms. The greater the ischaemia on stress echocardiography or invasive physiology, the greater the angina relief from PCI. Armed with this knowledge, clinicians can engage in a more informed and balanced discussion with patients, setting realistic expectations for what PCI is likely to achieve (Fig. 2).

## FUTURE DIRECTIONS

The overarching concept that a patient will only benefit from PCI if their symptoms are due to their stenosis, cannot be overstated, no matter how obvious this seems. For PCI to be effective as an antianginal procedure, it is our job to identify patients who truly have symptoms from epicardial disease. The ischaemic symptoms conferred by a stenosis have predictable characteristics. They are triggered by exertion and relieved by rest. The methods utilized in ORBITA-STAR directly mapped symptoms to stenosis, but relied on the necessary step of implanting a stent to repair any damaged coronary endothelium at the end of the experiment. This is clearly not possible for real-world decision-making. The challenge will be to find alternatives for lesion-specific balloon-inflation that do not mandate subsequent PCI. We need to find a way to interrogate the symptom-stenosis relationship without being forced to mechanically treat it. In addition, as our patients become increasingly comorbid with increased prevalence of multivessel coronary disease, it is also unclear how different stenoses within the same patient may contribute differentially to their overall angina. The ORBITA-MOON study is currently underway to answer this question [29]. Future work should aim to find strong predictors for the relationship between symptoms and stenosis, to arm interventionalists with the tools to decide if treating a stenosis will be meaningful.

## Acknowledgements

*None.*

### Financial support and sponsorship

*S.C. is a recipient of a Clinical Research Training Fellowship from NIHR [302493]. R.A.L. is a recipient of an Intermediate Research Fellowship from the British Heart Foundation [FS/ICRF/22/26051].*

### Conflicts of interest

*S.C. reports no conflicts of interest. KC reports no conflicts of interest. R.A.L. reports advisory board: Janssen Pharmaceuticals, Abbott, Philips, and speaker's honoraria: Abbott, Philips, Medtronic, Servier, Omniprex, Menarini.*

## REFERENCES AND RECOMMENDED READING

Papers of particular interest, published within the annual period of review, have been highlighted as:

▪ of special interest
▪▪ of outstanding interest
